# Intracellular biosynthesis of lipids and cholesterol by Scap and Insig in mesenchymal cells regulates long bone growth and chondrocyte homeostasis

**DOI:** 10.1242/dev.162396

**Published:** 2018-07-09

**Authors:** Hidetoshi Tsushima, Yuning J. Tang, Vijitha Puviindran, Shu-Hsuan Claire Hsu, Puviindran Nadesan, Chunying Yu, Hongyuan Zhang, Anthony J. Mirando, Matthew J. Hilton, Benjamin A. Alman

**Affiliations:** 1 Department of Orthopaedic Surgery and Regeneration Next Initiative, Duke University, Durham, NC 27710, USA; 2 Program in Developmental and Stem Cell Biology, The Hospital for Sick Children, Toronto, ON M5G 1X8, Canada

**Keywords:** Cholesterol, Chondrocyte, Scap, Enchodral, Insig

## Abstract

During enchondral ossification, mesenchymal cells express genes regulating the intracellular biosynthesis of cholesterol and lipids. Here, we have investigated conditional deletion of *Scap* or of *Insig1* and *Insig2* (Scap inhibits intracellular biosynthesis and Insig proteins activate intracellular biosynthesis). Mesenchymal condensation and chondrogenesis was disrupted in mice lacking *Scap* in mesenchymal progenitors, whereas mice lacking the Insig genes in mesenchymal progenitors had short limbs, but normal chondrogenesis. Mice lacking *Scap* in chondrocytes showed severe dwarfism, with ectopic hypertrophic cells, whereas deletion of Insig genes in chondrocytes caused a mild dwarfism and shortening of the hypertrophic zone. *In vitro* studies showed that intracellular cholesterol in chondrocytes can derive from exogenous and endogenous sources, but that exogenous sources cannot completely overcome the phenotypic effect of *Scap* deficiency. Genes encoding cholesterol biosynthetic proteins are regulated by Hedgehog (Hh) signaling, and Hh signaling is also regulated by intracellular cholesterol in chondrocytes, suggesting a feedback loop in chondrocyte differentiation. Precise regulation of intracellular biosynthesis is required for chondrocyte homeostasis and long bone growth, and these data support pharmacological modulation of cholesterol biosynthesis as a therapy for select cartilage pathologies.

## Abstract (precis)

**Summary:** Conditional deletion of genes that regulate intracellular cholesterol biosynthesis in mesenchymal cells or chondrocytes shows that precise regulation of biosynthesis is required for chondrocyte homeostasis and long bone growth.

## INTRODUCTION

Long bones grow through endochondral ossification, a coordinated process in which mesenchymal progenitor cells condense and differentiate into chondrocytes (Kronenberg, 2003). The chondrocytes proliferate, generate columnar structures and undergo hypertrophy, ultimately resulting in new bone formation and longitudinal growth. Cholesterol plays an important role in skeletal development, as inborn errors of metabolism inhibiting cholesterol synthesis are associated with skeletal dysplasias (Rossi et al., 2015). Experimental treatment with inhibitors of cholesterol biosynthesis also induces patterning defects, including pre-axial syndactyly or post-axial polydactyly (Gofflot et al., 2003), and reduced tibial growth plate height (Wu and De Luca, 2004). However, inborn errors of metabolism, and pharmacological inhibitors of cholesterol biosynthesis, alter cholesterol systemically in multiple cell types, confounding an understanding of the role of cholesterol synthesis in specific cell types. In addition to the skeletal manifestations of low cholesterol, high levels are associated with osteoarthritis, a form of cartilage degradation (Farnaghi et al., 2017). Hedgehog (Hh) signaling plays an important role in chondrocyte development and homeostasis (Lin et al., 2009). High levels of Hh signaling are associated with articular cartilage degeneration, and Hh signaling in these chondrocytes regulates intracellular cholesterol biosynthesis (Ali et al., 2016).

Intracellular cholesterol biosynthesis is regulated by proteins in the endoplasmic reticulum (ER), including sterol regulatory element-binding proteins (SREBPs), SREBP cleavage-activating protein (SCAP) and insulin-induced gene protein (INSIG). SCAP forms a complex with SREBP and functions as a sterol sensor (Brown and Goldstein, 1999). When cholesterol levels are low, SCAP escorts SREBP to the Golgi where proteases cleave SREBP to release the N-terminal domain of SREBP to traffic to the nucleus. In the nucleus, SREBP activates target genes for the biosynthesis of cholesterol. Conversely, when intracellular cholesterol levels are high, INSIG proteins prevent cholesterol biosynthesis by tethering the SREBP and SCAP complex to the ER membrane. The deletion of *Scap* inhibits cholesterol production in the involved cells. There are two INSIG proteins with functional redundancy. Deletion of both *Insig1* and *Insig2* increases intracellular cholesterol biosythesis. The relationship between systemic cholesterol levels and intracellular biosynthesis is complex. Plasma levels may not be related to intracellular levels, or intracellular levels to intracellular biosynthesis activity (August et al., 2007; Dietschy, 1998; Liscum and Underwood, 1995).

To elucidate the role of intracellular cholesterol biosynthesis within mesenchymal cells and chondrocytes in skeletal development, we focused on intracellular regulators of cholesterol biosynthesis using transgenic mice. Here, we have analyzed *Scap*-deficient mice and *Insig1*- and *Insig2*-deficient mice to study the role of intracellular cholesterol and lipid production in mesenchymal progenitor cells and chondrocytes. We found that cholesterol and lipid intracellular biosynthesis is a crucial process in chondrocyte development and homeostasis.

## RESULTS

### *Scap* is required for normal mesenchymal condensation

**Fig. 1.**
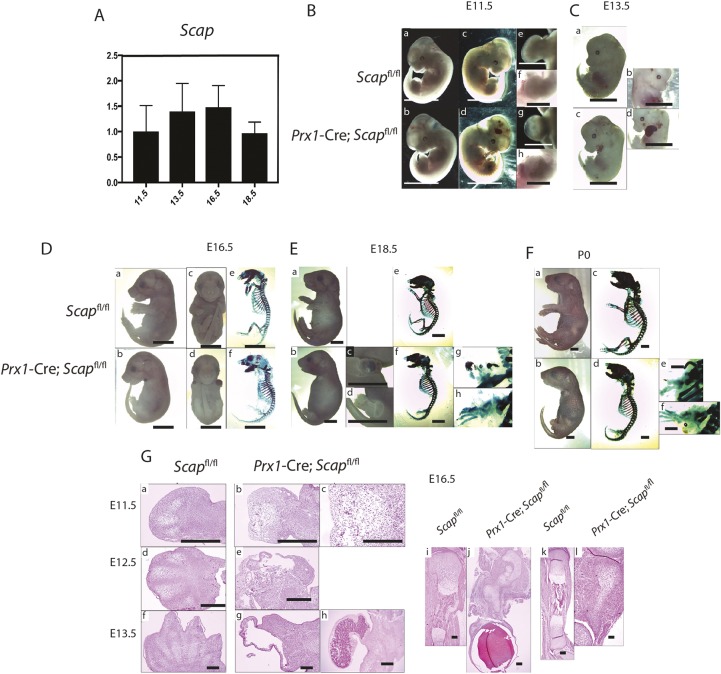
**Phenotype of mouse embryos lacking *Scap* in mesenchymal cells.** (A) RT-PCR data for *Scap* expression in microdissected limb buds from embryos at different stages showing that *Scap* is expressed during multiple stages of limb development (*n*=5 for each time point, means and 95% confidence intervals are shown). (B-F) Representative phenotype of embryos and mice in which *Scap* is inactivated in *Prx1*-expressing cells. (B) E11.5 embryos. b, d, g and h are embryos lacking *Scap* in *Prx1-*expressing cells, whereas the remainder of the images are controls. a, b, e and g are backlit photographs of the embryos in c, d, f and h. e-h are magnified views of the forelimb bud. The limb bud from embryos lacking *Scap* is rounder and contains a small hematoma compared with controls (*n*=8 for mutants, 7 for controls). (C) E13.5 embryos, showing a hematoma in the limb bud in embryos lacking *Scap* in *Prx1*-expressing cells. Three out of 11 embryos showed a phenotype as in d with a large hematoma encompassing the entire limb bud (*n*=6 for mutants, 8 for controls). (D) E16.5 embryos. a and b are side views, c and d are front views, and e and f are skeletal preparations. There is a short limb with malformed skeletal elements, which is more severe in the forelimbs (*n*=7 for mutants, 6 for controls). (E) E18.5 embryos. c, d, g and h show magnified views of the upper (c,g) and lower (g,h) extremities (*n*=6 for mutants, 7 for controls). (F) Mice at P0. A magnified view of the upper extremity is shown in e and the lower extremity in f (*n*=4 for mutants, 6 for controls). (G) Hematoxylin and Eosin staining of E11.5 to E16.5 limbs. E11.5 embryos are shown in a-c. Low- (b) and high- (c) magnification views of the forelimb bud from an embryo lacking *Scap* in *Prx1*-expressing cells showing disorganized mesodermal differentiation. E12.5 embryos are shown in d and e, showing the formation of a vascular cyst in the limb bud from an embryo lacking *Scap* in *Prx1*-expressing cells in e. E13.5 embryos are shown in f-h. A large cyst was found in four out of the six, as shown in g, whereas in two embryos there was an organized hematoma, as shown in h. The humerus at E16.5 (i,j) and the tibia (k,l) showing a substantial lack of cartilage in the limbs from embryos in which *Scap* is inactivated in *Prx1*-expressing cells, and a hematoma at the distal aspect of the forearm. Scale bars: 4 mm in Ba-Bd,C-F; 2 mm in Be-Bh; 1 mm in G.

We first examined the expression of *Scap* in in mesenchymal precursors. Studies of microdissected limb buds showed that *Scap* is expressed throughout embryonic development (Fig. 1A). To determine the effect of intracellular cholesterol and lipid biosynthesis in mesenchymal precursors, *Scap* was depleted in early limb bud mesenchyme by crossing *Scap*^f/f^ mice with *Prx1-*Cre mice. *Prx1* is expressed in limb bud cells that give rise to mesenchymal cells (Logan et al., 2002). The limb buds of mice lacking *Scap* were shorter and contained smaller mesenchymal condensations than controls (Fig. 1B,C,G). A hematoma was observed in many of the forelimbs. Later in development, there was severe forelimb shortening, without normal digit separation (Fig. 1D,E,F). At E18.5, the differences became more apparent with arrested forelimb development. Although the hind limbs were not as severely affected, they were also shorter than controls (Fig. 1F). At P0, both the forelimb and hindlimb showed a very small area of mineralization. Histological analysis confirmed the changes observed in the skeletal preparations (Fig. 1G).

### *Scap* regulates mesenchymal cell proliferation and differentiation to chondrocytes

**Fig. 2.**
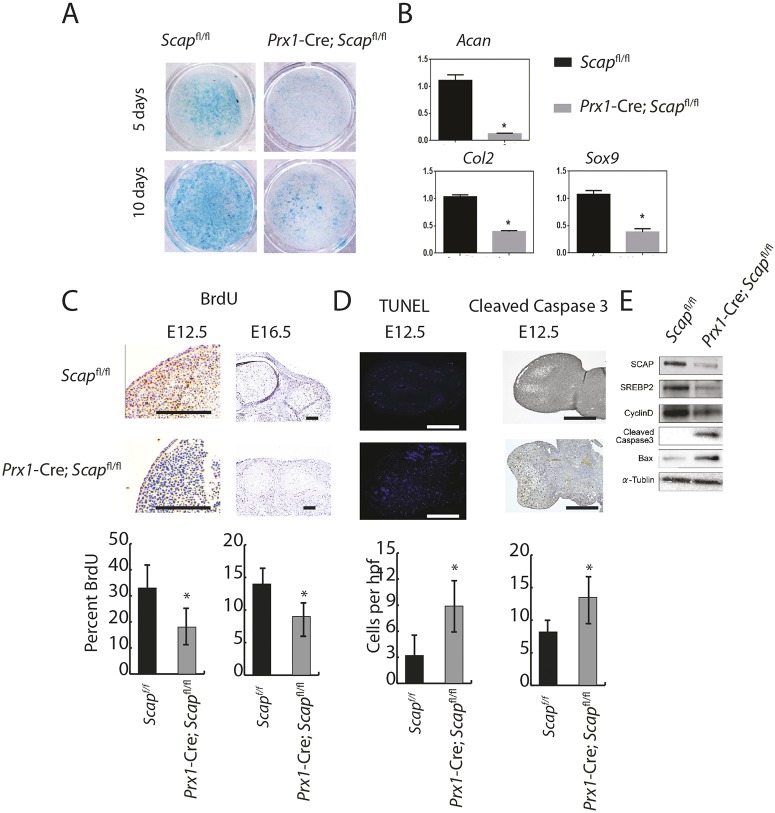
***Scap* regulates mesenchymal cell proliferation and differentiation.** (A) Representative Alcian Blue staining from micromass cultures showing decreased glycosaminoglycan production in limbs from mice with inactivation of *Scap* in *Prx1*-expressing cells (*n*=5 mutant and 5 controls). (B) Relative RNA expression comparing micromass cultures lacking *Scap* in *Prx1*-expressing cells with controls, showing decreased expression of markers of chondrogenesis at 10 days (*n*=6 mutant and 6 controls). Means and 95% confidence intervals are shown. (C) BrdU uptake in limbs. Graphs of means and 95% confidence intervals are underneath, *n*=6 for each time point and condition. (D) *Scap*-deficient mice contained TUNEL-stained cells and cleaved caspase 3 in the central regions of the developing bone, whereas TUNEL-stained cells were restricted to the margins of digital rays (*n*=6 for each genotype). Graphs of means and 95% confidence intervals are underneath, *n*=6 for each, *P*<0.05 for each time point and condition. (E) Representative western blot for cyclin D1, caspase 3 and Bax (*P<0.05). Scale bars: 1 mm.

Embryonic limbs from *Scap*-deficient mice contained fewer mesenchymal cells and there was decreased chondrogenesis. To determine whether this observed phenotype was due to intrinsic changes in cells differentiating to chondrocytes or to the chondrocytes themselves, or to both, we undertook micromass cultures. These cultures from *Scap*-deficient cells showed less Alcian Blue staining (Fig. 2A), consistent with a differentiation defect. Expression analysis showed that *Acan*, *Col2a1* and *Sox9* were strongly downregulated in the mutant limbs (Fig. 2B). Decreased cell proliferation or increased apoptosis, or both, might also explain the observed limb phenotype. BrdU staining in the limbs showed a reduction in the number of positively stained cells in mutant mice (Fig. 2C). TUNEL-positive and cleaved caspase 3-positive cells existed in interdigital spaces at E12.5 in wild-type mice, but TUNEL-positive and cleaved caspase 3-positive cells were noted throughout the limb in mutant animals. Western analysis also showed that cyclin D1 was strongly downregulated in the mutant limbs and cleaved caspase 3 and Bax was upregulated in the mutant limb (Fig. 2D,E). Thus, *Scap* is required for multiple processes necessary for enchondral growth, including differentiation to chondrocytes, the maintenance of cell proliferation and the prevention of ectopic apoptosis.

### Loss of *Scap* in chondrocytes results in a disordered growth plate

**Fig. 3.**
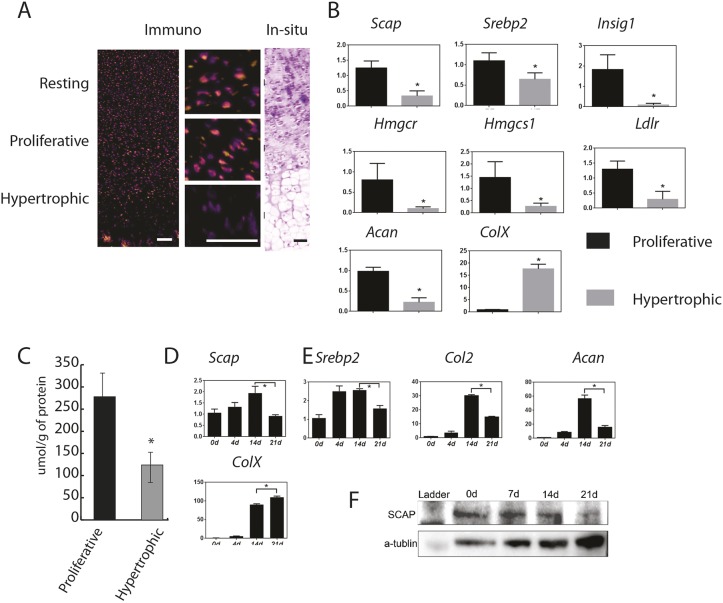
**Scap expression and cholesterol levels are decreased in hypertrophic chondrocytes*.*** (A) Immunofluorescent staining and *in situ* hybridization of E16.5 embryos showed that Scap protein was expressed in the round cell zone (resting) and proliferation zone, but was decreased in the hypertrophic zone. Left panel is immunofluorescent staining, middle panels are magnified views of the three zones and the right panels show *in situ* hybridization. Scale bars: 100 μm. (B) RT-PCR data from microdissected regions of the proliferating zone (PZ) and hypetrophic zone (HZ) of the growth plate showing differential regulation of *Scap* and other genes involved in intracellular biosynthesis of cholesterol and lipids (from E16.5 limbs, *n*=8 in each group). Black represents controls; gray represents cells from limbs lacking *Scap*. (C) Cholesterol levels in the proliferating zone (PZ) and hypetrophic zone (HZ) of the growth plate (*n*=4). (D,E) Scap expression micromass cultures also showed that *Scap* expression was gradually decreased over time as cells progressed through chondrocytic differentiation. D shows RNA data (*n*=8) and E a representative western blot. Means and 95% confidence intervals are shown (*P<0.05).

We next examined *Scap* expression in growth plate chondrocytes. Immunofluorescent staining and *in situ* hybridization from E16.5 embryo distal femurs showed that Scap protein was expressed in round/resting cell zone (RZ) and proliferation zone (PZ), but its level of expression was lower in the hypertrophic zone (HZ) (Fig. 3A). To confirm the changes in *Scap* expression during chondrocyte hypertrophy, we microdissected growth plate cells into the PZ and HZ, and extracted mRNA. *Scap* expression, as well as multiple other genes involved in cholesterol biosynthesis, were decreased in HZ chondrocytes (Fig. 3B). Cholesterol levels were also lower in the HZ cells (Fig. 3C). Micromass cultures showed that *Scap* expression was decreased as chondrocytes differentiated as well (Fig. 3D). The results of western analysis were consistent with that of QPCR (Fig. 3E).

**Fig. 4.**
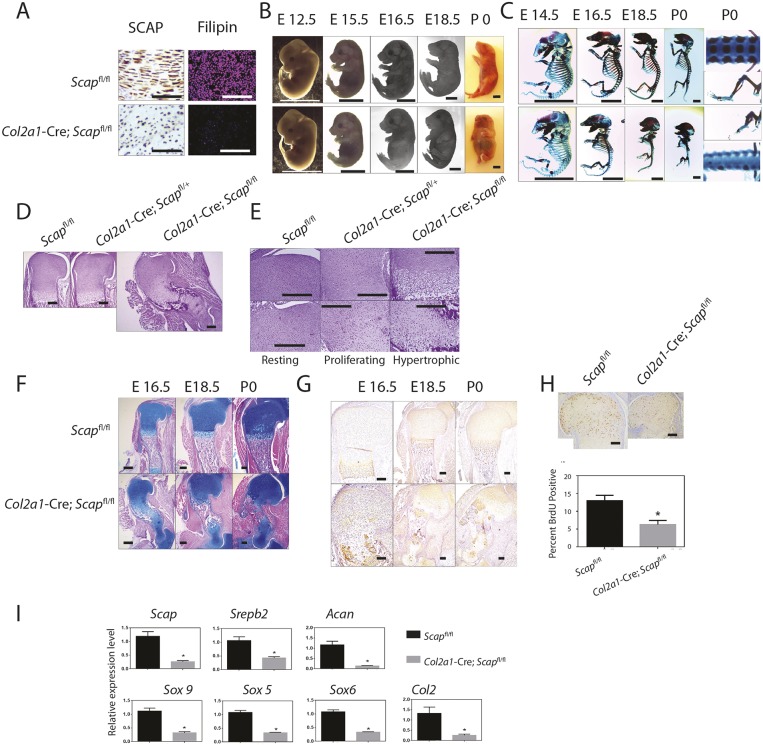
**Loss of Scap in chondrocytes results in a disordered growth plate and severe dwarfism.** (A) Immunohistochemistry for SCAP (left) and filipin fluorescent staining (right) for control limbs (top) and for limbs from mice lacking *Scap* in *Col2a1*-expressing cells (bottom), confirming lack of SCAP expression and decreased intracellular cholesterol and lipids in mutant mice. (B,C) Whole-mount and skeletal preparations of representative E12.5 to P0 mice. Top rows show mice expressing *Scap* in *Col2a1*-expressing cells; the bottom rows show mice lacking *Scap* in these cells (*n*>5 for each genotype at each age). P0 images of the spine and limb show a lack of spinal cartilage development and severely shortened long bones. (D) Hematoxylin and Eosin staining of fetal limbs of E16.5 humerus sections with control limbs on the right, limbs from a mouse lacking *Scap* in one allele in the middle and limbs from a mouse lacking *Scap* in both alleles in *Col2a1*-expressing cells on the left. (E) Magnified views of 16.5 humeri showing views of the resting, proliferating and hypertrophic zones. The mutant limb demonstrates ectopic hypertrophic cells. (F) Alcian Blue staining of the upper limb. (G) Type X collagen staining of the upper limb. (H) BrdU incorporation, with the limbs from mice lacking *Scap*. (I) Expression of various genes in the mutant and control limbs. Means and 95% confidence intervals are shown (*P<0.05). Scale bars: 250 μm in A; 4 mm in B,C; 500 μm in D-H.

To determine the chondrocyte-autonomous role of *Scap in vivo*, we generated mice in which *Scap^f/f^* conditional deletion was driven by the regulatory elements of type II collagen (Fig. 4A). *Scap*-deficient fetal mice were shorter than control littermates, had a foreshortened snout, a rounded skull, a short tail, severe dwarfism of the limbs and protruding abdomens. The shortening of limbs was not observed at E12.5, but was obvious at E15.5 (Fig. 4B,C). Whole-mount staining showed that the *Scap*-deficient mice had a primitive ribcage, a shorter sternum and shorter ribs, with delayed mineralization of the vertebrae. Mice lacking both *Scap* alleles within chondrocytes died at birth. Examination of the growth plates at E16.5, showed less primary ossification, with a disorganized round cell zone (Fig. 4D,E). There was a disrupted columnar structure in the proliferation zone, and a reduced number of hypertrophic chondrocytes (Fig. 4E). The hypertrophic zone was small and disordered, and there was decreased expression of the hypertrophic marker, type X collagen (Fig. 4F,G). The proportion of BrdU-incorporating cells was lower (Fig. 4H). There was also decreased expression of genes expressed by chondrocytes, such as *Acan*, *Col2a1* and *Sox9* (Fig. 4I).

### The phenotype of *Scap*-deficient chondrocytes cannot be fully rescued by exogenous cholesterol

**Fig. 5.**
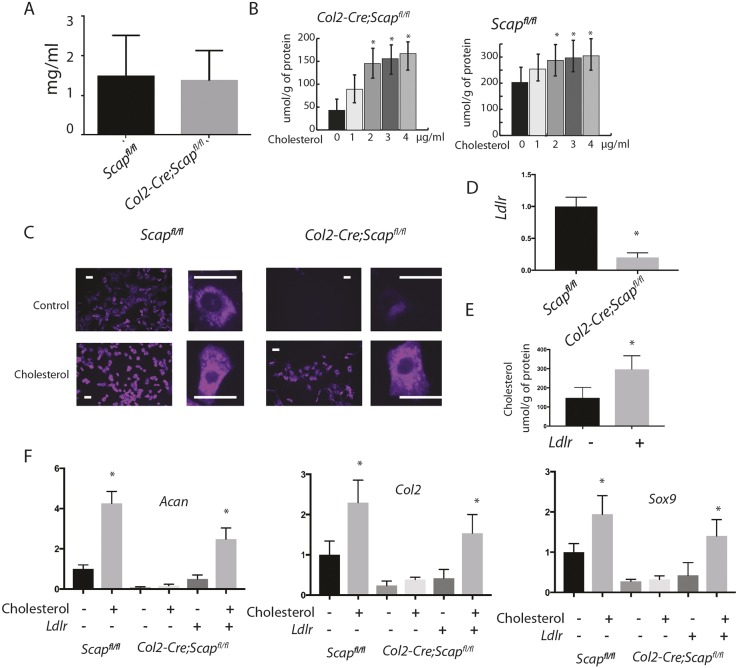
**Exogenous cholesterol does not change the phenotype of chondrocytes lacking *Scap* in Col2-expressing cells.** (A) Serum cholesterol levels in mice lacking *Scap* in chondrocytes at P0, showing no difference between serum cholesterol levels between *Scap*-deficient and control mice (*n*=5 in each group). (B) Intracellular cholesterol levels measured using mass spectroscopy in chondrocyte cultures from *Scap*-deficient and control mice (*n*=8 at each time point for each genotype). Cholesterol levels increase with cholesterol supplementation, but in *Scap*-deficient cells this did not reach the same level as observed in control cells. (C) Filipin staining showing that treatment with cholesterol increases staining in cultures, but cholesterol supplementation cannot increase the levels in *Scap*-deficient cells to levels observed in controls. High-power magnification shows intracellular localization. Cholesterol is primarily located in the cytoplasm in these cells, regardless of the genotype. Scale bars: 20 μm. (D) Overexpression of *Ldlr* in *Scap*-deficient chondrocytes increases intracellular cholesterol. (E) Cholesterol levels increase with overexpression of *Ldlr*. (F) RT-PCR for expression of *Acan*, *Col2a1* and *Sox9* in cultures from *Scap*-deficient and control mice, with or without cholesterol supplementation or overexpression, or overexpression of *Ldlr* (*n*=5 in each group). Data are mean with 95% confidence levels (*P<0.05 compared with control, i.e. each genotype without cholesterol supplementation or *Ldlr* overexpression).

To investigate the relative contribution of intracellular cholesterol synthesis and extracellular cholesterol, we first analyzed serum and intracellular cholesterol levels in the mice lacking Scap in *Col2a1*-expressing cells. There was not a difference in serum cholesterol level between *Scap*-deficient and control mice (Fig. 5A). We then examined whether exogenous cholesterol could reverse the changes in primary chondrocyte cultures from mice lacking *Scap* in collagen 2-expressing cells. Treatment of control chondrocytes with exogenous cholesterol increased intracellular levels, but reached a threshold at 2 μg/ml. Treatment with 3 μg/ml was thus used for the remainder of the studies. The level of intracellular cholesterol in *Scap*-deficient chondrocytes did not reach that of controls (Fig. 5B). Filipin is a naturally fluorescent polyene antibiotic that binds to unesterified cholesterol (Bornig and Geyer, 1974). Staining for filipin was substantially decreased in cells lacking *Scap* expression. Treating cells with cholesterol increased staining, but not to the level observed in cells expressing *Scap*. Although the amount of staining increased, the intracellular localization of cholesterol did not differ between exogenous and endogenous sources, and staining was located primarily in the cytoplasm (Fig. 5C). Interestingly, *Ldlr* expression is low in chondrocytes lacking *Scap* (Fig. 5D), and the product of this gene can regulate the ability of cells to uptake extracellular cholesterol (Brown et al., 1983; Zhang et al., 2016). Thus, we examined whether overexpression of *Ldlr* could increase intracellular cholesterol level. In response to overexpression of Ldlr, the level of intracellular cholesterol approached control cell levels with exogenous cholesterol supplementation (Fig. 5E). Although exogenous cholesterol increased the expression of *Acan*, *Col2* and *Sox9* expression in control mice, in mutant chondrocytes, exogenous cholesterol did not significant alter expression of these genes. However, in *Scap*-deficient cells in which *Ldlr* was also overexpressed, the level of expression of these genes increased, but not nearly to the level observed in cells expressing *Scap* (Fig. 5F). These results suggest that exogenous cholesterol is insufficient to maintain chondrocyte homeostasis in the absence of either intracellular biosynthesis or overexpression of *Ldlr*. Our data are also consistent with the notion that overexpression of *Ldlr* is not as effective as intracellular biosynthesis at maintaining the physiological function of chondrocyte.

### Deletion of Insig genes cause dwarfism

**Fig. 6.**
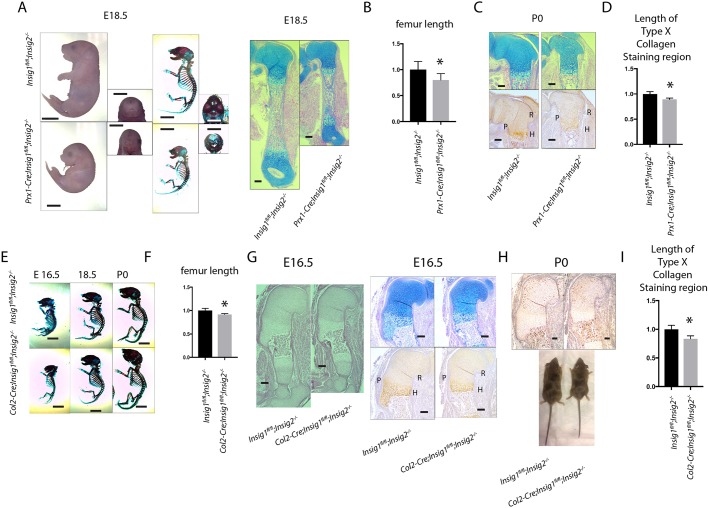
**Deletion of Insig genes within mesenchymal cells or chondrocytes causes dwarfism.** (A) Representative whole-mount, skeleton and long bone histology of E18.5 embryos and mice, showing dwarfism but intact overall bone structure when comparing mice lacking both Insig genes in *Prx1*-expressing cells with controls. (B) Femur length of E18.5 *Prx1-cre*; *Insig1^flox/flox^; Insig2^−/−^* and *Insig1^flox/flox^; Insig2^−/−^* embryos. The length of *Insig1^flox/flox^; Insig2^−/−^* is normalized to 1 (*n*=8 for *Prx1-cre*; *Insig1^flox/flox^; Insig2^−/−^* and 9 for *Insig1^flox/flox^; Insig2^−/−^*). Data are means with 95% confidence intervals. *P<0.05 compared with data from *Insig1^flox/flox^; Insig2^−/−^*. (C) The histology of the humeral growth plate at P0. R, resting zone; P, proliferative zone; H, hypertrophic zone. Bottom panels show type X collagen staining labeling the hypertrophic zone. (D) Relative length of type X collagen-expressing cells with the length of the growth plates in C. *Insig1^flox/flox^; Insig2^−/−^* is normalized to 1 (*n*=6 for *Prx1-cre*; *Insig1^flox/flox^; Insig2^−/−^* and 5 for *Insig1^flox/flox^; Insig2^−/−^*). Data are mean with 95% confidence intervals (*P<0.05 compared with data from *Insig1^flox/flox^; Insig2^−/−^*). (E) Representative skeletons of embryos and mice showing dwarfism in mice lacking both Insig genes in *Col2a1*-expressing cells, showing intact overall bone structure compared with controls. (F) Femur length of P0 *Col2-cre*; *Insig1^flox/flox^; Insig2^−/−^* and *Insig1^flox/flox^; Insig2^−/−^* embryos. The length of *Insig1^flox/flox^; Insig2^−/−^* is normalized to 1 (*n*=7 for *Col2-cre*; *Insig1^flox/flox^; Insig2^−/−^* and 7 for *Insig1^flox/flox^; Insig2^−/−^*). Data are mean with 95% confidence intervals (*P<0.05 compared with data from *Insig1^flox/flox^; Insig2^−/−^*. (G) Representative histology of an E16.5 humerus. Alcian Blue and type X collagen staining of the same limbs are shown. R, resting zone; P, proliferative zone; H, hypertrophic zone. (H) Representative images of mice at P0. (I) Relative length of type X collagen-expressing cells with the length of the growth plates in P0 limbs. *Insig1^flox/flox^; Insig2^−/−^* is normalized to 1 (*n*=5 for *Col2-cre*; *Insig1^flox/flox^; Insig2^−/−^* and 6 for *Insig1^flox/flox^; Insig2^−/−^*). Data are means with 95% confidence intervals (*P<0.05 compared with data from *Insig1^flox/flox^; Insig2^−/−^*). Scale bars: 4 mm in A,E; 1 mm in C,G,H.

Because a lack of intracellular cholesterol production caused such a dramatic phenotype, we examined whether overproduction of intracellular cholesterol affected skeletal development. *Insig1* and *Insig2* (Engelking et al., 2005) encode proteins with redundant functions. Reduction of both INSIG proteins leads to an increase in nuclear SREBPs and production of cholesterol. Although the articular chondrocyte phenotype of mice lacking both Insig genes has been previously reported (Ali et al., 2016), the developmental phenotype has not been analyzed. *Insig1^flox/flox^; Insig2^−/−^; Prx1cre* mice were generated and compared with control mice. Mutant mice were viable and showed dwarfism and a cleft palate (Fig. 6A-D), and exhibited postnatal lethality. Next, we compared the phenotype of *Insig1 ^f/f^; Insig 2^−/−^; Col2cre* with that of *Insig1 ^f/f^; Insig 2^−/−^* control mice. The length of Type X collagen staining was decreased in mice lacking both Insig genes in chondrocytes (Fig. 6E-I). The mutant mice had shorter limbs than those of controls. Although a lack of intracellular cholesterol and lipid biosynthesis produced a more robust phenotype than did increasing cholesterol levels, we found a subtle phenotype, illustrating that intracellular cholesterol biosynthesis needs to be maintained at an optimal level for normal chondrocyte function.

### Hedgehog signaling and intracellular cholesterol production regulate each other during chondrocyte differentiation

**Fig. 7.**
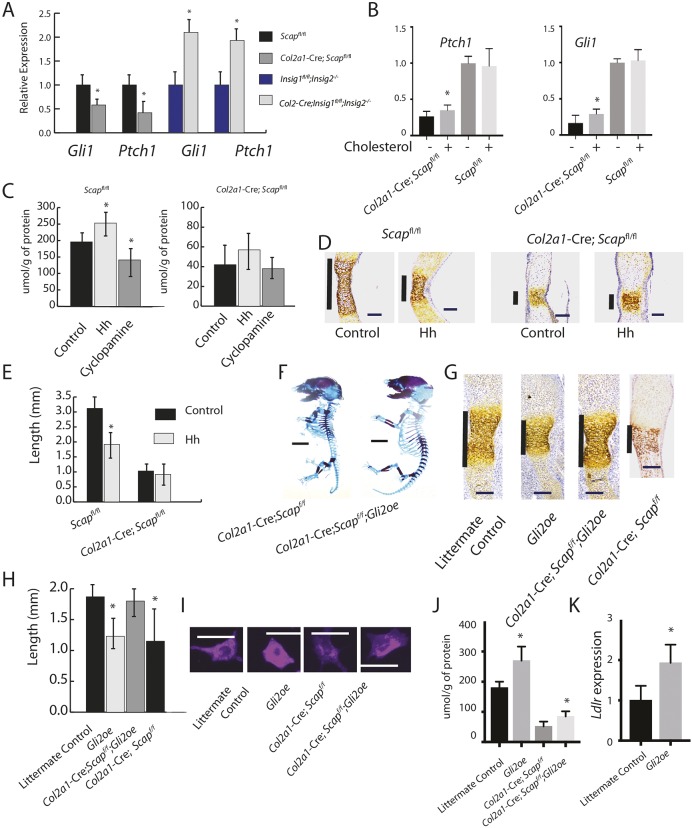
**Hedgehog and cholesterol regulating each other in the growth plate.** (A) Expression of *Ihh* and Hedgehog target genes in cells from mice lacking *Scap* or the Insig genes in *Col2*-expressing cells and controls (*n*=6 for each genotype). Expression in littermate controls was arbitrarily normalized to 1. (B) Effect of treatment with 3 μg/ml exogenous cholesterol on Hh target gene expression on chondrocyte cultures from mice lacking *Scap* in chondrocytes or littermate controls. Data are mean with 95% confidence intervals (*P<0.05). Data are normalized so that expression in littermate controls averages 1 (*n*=5 in each group). (C) Cholesterol levels in metatarsal explants treated with Ihh or control. (D) Treatment with the Ihh-N ligand in mice lacking *Scap* in *Col2*-expressing cells resulted in a level of type X collagen expression similar to that seen in control explants. Vertical line shows the length of type X collagen-expressing cells. (E) Graph shows the length of collagen type X collagen-expressing cells in the explants (*n*=5 in each group). (F) Crossing mice lacking *Scap* in *Col2a1*-expressing cells with mice overexpressing *Gil2* in in *Col2a1*-expressing cells, results in a partial rescue of the phenotype shown in E16.5 mice. (G) Metatarsals from E16.5 mice showing rescue of type X collagen-expressing cells in mice overexpressing Gli2 by depleting *Scap*. Vertical line shows the length of type X collagen-expressing cells. (H) Length of type X collagen-expressing cells. Data are means with 95% confidence intervals from samples in G (*P<0.05). *n*>8 for each genotype at each time point. (I) Filipin staining of representative chondrocytes from mice in G. (J) Cholesterol level in cells. Data are means with 95% confidence intervals (*P<0.05). *n*=4 in each group. (K) *Ldlr* expression, with level in littermate controls normalized to 1. Data are mean with 95% confidence intervals (*P<0.05). *n*=6 in each group. Scale bars (horizontal lines): 1 mm in D,G; 4 mm in F; 20 μm in I.

Our data show that intracellular cholesterol needs to be maintained at an optimal level for normal growth plate chondrocyte differentiation and the same holds true for hedgehog signaling. As hedgehog signaling and intracellular cholesterol biosynthesis cholesterol can interact on several levels (Luchetti et al., 2016; Porter et al., 1996; Xiao et al., 2017), this raises the possibility that they could act in part through a feedback loop regulating chondrocyte differentiation. Ihh and its target genes were examined in primary chondrocytes from *Scap*- or *Insig*-deficient mice. There was a significant decrease in the expression of *Ihh* and its target genes, *Gli1* and *Ptch1*, in *Scap*-deficient limbs, whereas expression of the Hh-regulated genes were upregulated in *Insig*-deficient limbs (Fig. 7A). As cholesterol can modify Hh ligands or regulate its intracellular signaling, we examined primary chondrocyte cultures from Scap-deficient or control littermate mice. Treatment with 3 μg/ml exogenous cholesterol slightly increased Hh target genes in *Scap*-deficient cells, but had no effect on cells from littermate controls (Fig. 7B). This suggests that the differences in of Hh signaling levels between *Scap*-deficient, *Insig*-deficient and control littermate cells are more likely due to intracellular signaling changes than to extracellular changes in how cholesterol modifies Hh activity. Treatment with hedgehog ligand increased the cholesterol level, whereas treatment with cylopamine decreased the cholesterol level in organ cultures from E16.5 metatarsal bones, but there was little change in cholesterol levels in limbs lacking *Scap* (Fig. 7C). Next, we examined how Hh activation and cholesterol biosynthesis might interact in the regulation of chondrocyte differentiation. In control explant cultures, treatment with Hh ligand results in a smaller zone of type X collagen expression, a marker of hypertrophic chondrocytes. In contrast, this regulation is lost after treatment with Hh ligand in explants from limbs lacking *Scap* in chondrocytes (Fig. 7D,E). To determine the role of Hh signaling in the phenotype of *Scap* deletion, *Scap ^flox/flox^*; *Col2a1-Cre* mice were crossed with *Tg(Gli2; Col2a1)* mice, in which the Gli2 mediated Hh transcriptional activator is overexpression in *Col2*-expressing cells. As Gli2 is not efficiently processed to a repressor form, this constitutively activates Hh signaling downstream of Hh ligand activation (Hopyan et al., 2002). The overexpression of Gli2 partially rescued the short limb phenotype associated with *Scap* deficiency in chondrocytes, with a 27% increase in upper limb length and a 23% increase in lower limb length at E16.5 (Fig. 7F). Type X collagen expression in the E16.5 metatarsals was rescued by overexpression of Gli2 (Fig. 7G,H). Filipin staining and cholesterol levels showed that Gli2 overexpression increased cholesterol levels (Fig. 7I,J). Interestingly, there is an increase in cholesterol levels in cells lacking *Scap* with *Gli2* overexpression. One possibility is that Gli2 expression would increase cholesterol uptake and, indeed, *Ldrl* expression is increased in these cells (Fig. 7K).

## DISCUSSION

Here, we demonstrate the importance of genes regulating intracellular cholesterol and lipid biosynthesis in mesenchymal and chondrocyte differentiation. Mice lacking *Scap* in mesenchymal precursors showed reduced mesenchymal condensation and differentiation to chondrocytes, associated with reduced proliferation and increased apoptosis. Moreover, a balance in intracellular cholesterol and lipid synthesis is crucial to maintain normal chondrocyte differentiation, as illustrated by the phenotype observed in both *Scap*- and *Insig*-deficient animals. Interestingly, the mice have a normal serum cholesterol level and *in vitro* studies showed that there was only a mild rescue of the *Scap*-deficient phenotype with exogenous cholesterol treatment, even when intracellular levels reached that of controls. Thus, our data show that cell-autonomous intracellular cholesterol and lipid biosynthesis are crucial for normal enchondral ossification and maintenance of chondrocyte homeostasis.

Intracellular cholesterol and lipid synthesis regulates the differentiation, proliferation and apoptosis of undifferentiated mesenchymal cells in the developing limb. In the growth plate, *Scap* expression and intracellular cholesterol levels decrease with differentiation. *Insig1* is also differentially regulated during enchondral ossification (Li et al., 2016). As such, cholesterol and lipid biosynthesis regulate limb development and enchondral bone growth at multiple stages during limb development. LDL receptors provide a mechanism for delivery of cholesterol to cells (Brown et al., 1983). *Ldlr* expression is low in hypertrophic chondrocytes and in *Scap*-deficient chondrocytes. When *Ldlr* is overexpressed, intracellular cholesterol levels can reach those observed in chondrocytes expressing *Scap*. Intriguingly, however, this is insufficient to maintain expression of genes important in chondrocyte homeostasis. Thus, our data is also consistent with the notion that intracellular cholesterol in chondrocytes can derive from exogenous and endogenous sources, but that exogenous sources alone cannot overcome the phenotypic effect of *Scap* deficiency.

Previous work shows that Hh signaling regulates genes important for intracellular cholesterol and lipid synthesis in chondrocytes (Ali et al., 2016; Wu and De Luca, 2004). Furthermore, cholesterol can regulate Hh signaling at multiple levels in its signaling cascade, from ligand processing to modification of receptors and intracellular transducers of transcriptional activity (Huang et al., 2016; Luchetti et al., 2016; Porter et al., 1996). We found that *Scap* expression also regulates Hh transcriptional activity in chondrocytes. With differentiation, cholesterol and lipid biosynthesis decreases, which is associated with Hh signaling inhibition. This finding and our explant data are consistent with the notion that Hh and cholesterol biosynthesis regulate each other during chondrocyte differentiation. The addition of Hh did not completely rescue the *Scap*-deficient phenotype, whereas activation of Gli did rescue the *Scap*-deficient phenotype. In addition, exogenous cholesterol is not as effective in regulating Hh signaling as modulating intracellular biosynthesis. Although our findings are consistent with cholesterol modulating Hh signaling at multiple levels in the Hh signaling cascade, it suggests that Scap acts primarily intracellularly to transmit the Hh signaling to the Gli transcription factors. Cholesterol also regulates other signaling pathways, such as Wnt signaling (Sheng et al., 2014), that can regulate chondrocyte differentiation (Hulce et al., 2013). Thus, there are also multiple mechanisms by which cholesterol and Hh might interact in regulating differentiation during enchondral ossification.

The major site of cholesterol production is the liver (Turley, 2004). Yet mice lacking *Scap* in hepatocytes are relatively healthy (McFarlane et al., 2015). We found that mice lacking *Scap* in mesenchymal cells do not survive after birth. Our results in mesenchymal cells and chondrocytes are reminiscent of those found in the intestine, where depletion of *Scap* causes severe enteropathy and death (McFarlane et al., 2015). The proliferation and differentiation of chondrocytes requires multiple signaling pathways, and cholesterol and lipid biosynthesis can regulate many of these pathways (Incardona and Eaton, 2000; Mann and Beachy, 2000, 2004). Although exogenous cholesterol can cause changes in chondrocytes expressing *Scap*, this capacity is lost in chondrocytes lacking *Scap*. This suggests that the proliferation and differentiation of chondrocytes requires high levels of intracellular cholesterol and lipids that cannot be completely compensated for exogenously. The decreased levels of *Scap* in cells near terminal differentiation of the growth plate is consistent with the notion that these cells are slowing their metabolism and do not require such high levels of cholesterol and lipids. Furthermore, the Hh regulation of *Scap* expression raises the possibility that this signaling pathway maintains a balance of high cholesterol synthesis in the less differentiated cells, and as Hh activity declines, so does intracellular cholesterol biosynthesis.

Cholesterol biosynthesis can be pharmacologically targeted, and as such represents a therapeutic opportunity. Our data showing dwarfing with either activation or inactivation of this biosynthetic pathway are consistent with the notion that its level needs to be precisely regulated for normal bone growth. Such a notion is consistent with being part of a feedback loop regulating the pace of cell differentiation. Interestingly, such modulation has been shown to play a role modulating endochondral growth in achondroplasia (Yamashita et al., 2014). Although we do not have data on achondroplasia, it is intriguing to speculate that cholesterol level is deregulated in achondroplasia. This could explains how pharmacological manipulation of cholesterol synthesis improves bone growth in this condition. It is also possible that other conditions associated with abnormal bone growth might be treated in a similar manner. There are neoplastic processes, such as enchondromas and chondrosarcomas, that can arise from growth plate chondrocytes. The inhibition of cell proliferation by modulation of this biosynthetic pathway raises the possibility that pharmacologically targeting cholesterol synthesis could be developed into an effective therapeutic approach.

## MATERIALS AND METHODS

### Experimental animals

*Scap^flox/flox^* (B6;129-*Scap^tm1Mbjg^*/J) (Matsuda et al., 2001) and *Insig1^flox/flox^; Insig2^−/−^* (B6;129S6-*Insig1^tm1Mbjg^ Insig2^tm1Mbjg^*/J) mice (Engelking et al., 2005) were obtained from the Jackson laboratory. *Scap^flox/flox^* mice were crossed with *Col2a1-Cre* mice, producing mice conditionally lacking one or both alleles of *Scap*. Similarly, *Scap^flox/flox^* mice were crossed with *Prx1-Cre* mice. Likewise, we generated *Insig1^flox/flox^; Insig2^−/−^*; *Col2-cre* and *Insig1^flox/flox^; Insig2^−/−^*; *Prx1-cre.* We also used *Tg(Gli2;Col2a1)* mice, which are characterized by the expression of the Hh-activated growth factor Gli2 in growth plate chondrocytes driven by the regulatory elements of Col2 (Hopyan et al., 2002). These mice were crossed with *Scap^flox/flox^*;*Col2a1* mice to generate *Scap^flox/flox^*;*Col2a1-Cre;Tg(Gli2;Col2a1)* mice. All of the mice were on a B6 background and equal numbers of male and female embryos were examined. Mice were analyzed after whole-mount staining using Alcian Blue and Alizarin Red, using Hematoxylin and Eosin staining, and using Safranin O staining and immunohistochemistry. All animals were used according to the approved protocol by Institutional Animal Care and Use committee of Duke University.

### Micromass cultures of limb mesenchyme

Mouse embryos at E12.5 were harvested and fore- and hindlimb buds collected. Limb buds were incubated in 0.25% trypsin at 4°C for 1 h. Cells were placed (1×10^5^ cells) in the center of the culture dish and allowed cells to attach for 3 h. DMEM F/12 (Invitrogen) supplemented with 10% fetal bovine serum (FBS) (Gibco), and 1% penicillin and streptomycin were gently added to the plate. Cells were incubated for various time periods, after which expression analysis or Alcian Blue staining was performed.

### Analysis of gene expression

Total RNA was extracted from cells or tissues using RNA easy mini kits (Qiagen) according to the manufacturer's instructions. Total RNA was reverse transcribed in PrimeScript RT Reagent Kit with gDNA Eraser (Perfect Real Time) (Takara Bio) to make single-stranded cDNA. Quantitative real-time RT-PCR (Biorad) was performed using SYBR Premix Ex Taq II (Tli RNase H Plus) (Takara Bio). Analysis of gene expression was performed using the ΔΔCt method. Data were normalized to expression of the HPRT mRNA levels. Each experiment was performed in triplicate.

### Western blotting

Proteins were extracted from cultured cells using RIPA Lysis and Extraction Buffer (Pierce) with Halt Protease and Phosphatase Inhibitor Cocktail (Pierce). The samples were centrifuged at 18,800 ***g*** for 15 min at 4°C. The BCA colorimetric method was performed to check the concentration of the proteins. Protein samples (20 μg) were electrophoresed using NuPAGE 4-12% Bis-Tris gel electrophoresis (Invitrogen). The gels were transferred to an Immune-Blot PVDF Membrane (Biorad) in Tris/glycine buffer (pH 8.3) containing 20% methanol. After blocking nonspecific binding sites with 4% nonfat milk or 4% BSA in 0.1% Tween 20/phosphate-buffered saline or Tris-buffered saline (PBS-T or TBS-T) for 1 h, the membranes were treated at 4°C overnight with the following primary antibodies in blocking buffer: polyclonal rabbit anti-Scap antibody (1:200, PA5-28982, Thermo Scientific), polyclonal rabbit anti-SREBP2 antibody (1:400, ab30682, Abcam), rabbit polyclonal anti-cyclin D antibody (1:1000, 2978, Cell Signaling Technology), polyclonal rabbit anti-cleaved caspase 3 antibody (1:1000, 9664, CST) and polyclonal rabbit anti-Bax antibody (1:1000, 2772, CST). After washing, horseradish peroxidase-conjugated secondary antibody (1:1000, AMI4404, BioSource International) was added for 1 h at room temperature. The immune reactive blots were detected using ECL Plus (Amersham Pharmacia Biotech).

### Immunohistochemistry

Samples were fixed in 10% neutral buffered formalin or 4% paraformaldehyde and decalcification performed in formic acid or 14% EDTA. Antigen retrieval was carried out using Proteinase K for 15 min. Endogenous peroxidase activity was a 30 min block and then incubated overnight at 4°C with primary anti-SCAP antibody (1:200, PA5-28982, Thermo Scientific), anti-sox9 antibody (1:1000, AB5535, Merck Millipore), anti-Col X (1:800, LSL-LB-0092, LSL Cosmo Bio) or anti-MMP13 (1:1000, MS-825-P0, Thermo Scientific). Finally, the samples were counterstained with Hematoxylin.

### Proliferation and apoptosis analysis

DNA synthesis of cells was assayed by bromodeoxyuridine (BrdU). Pregnant females were intraperitoneally injected with BrdU 100 mg/kg. A BrdU Staining Kit (Invitrogen) was used for analysis. Detection of apoptosis was performed by TUNEL staining using the *In Situ* Apoptosis Detection Kit (Takara Bio) according the manufacture's protocol.

### Primary culture of costal chondrocytes from mice

E18.5 and P3-P5 neonatal pups were used for primary chondrocyte cultures as described previously (Gosset et al., 2008). The sternum and ribs were washed with PBS and digested in 15 ml of 2 mg/ml pronase (Roche) at 37°C for 1 h with constant agitation. Ribs were washed three times with PBS and digested in 3 mg/ml of collagenase D (Plaisant et al., 2009) in a 37°C humidified cell culture chamber for 1 h. After placing in fresh collagenase D, the ribs were transferred to a petri dish and incubated at 37°C in a humidified cell culture chamber for 4-6 hours and filtered using a 45 μm cell strainer.

### Ihh treatment and overexpression of *Ldlr*

To overexpress *Lrlr*, cells were infected with Lenti-CMV and Lenti-CMV-Ldlr virus (LDLR Lentivirus, Mouse, from Applied Biological Materials, LVP536261). After 24 h, the cells were treated with puromycin and then studied with and without cholesterol (water soluble, Sigma C4951) added to the media for 24 h. IHH recombinant protein (1705-HH-025 R&D systems) was used at a concentration of 250 ng/ml, as in previous studies (Veistinen et al., 2017).

### Metatarsal organ cultures

E16.5 mouse embryos were prepared. Three central metatarsal bones (2nd, 3rd and 4th digit) were dissected from the hind limbs of the embryos and were placed in 24-well plates in 300 μl of α-MEM (Invitrogen) supplemented with 50 μg/ml ascorbic acid, 1 mM β-glycerophosphate, and 0.2% bovine serum albumin. Explants were grown at 37°C in a humidified 5% CO_2_ incubator. The medium was changed every 2 days. The cultured rudiments were harvested on day 5 and then fixed in fresh 10% neutral buffer formalin (NBF) overnight at room temperature. Each metatarsal bone obtained from identical mouse embryos were transfected with adenovirus vectors expressing SCAP or GFP control and cultured at 37°C in a humidified 5% CO_2_ incubator for 4 days. Safranin O and immunohistochemical staining was performed.

### Cholesterol supplementation and intracellular cholesterol level analysis

To supplement media, between 1 and 4 μg/mg of cholesterol (Sigma-Aldrich) was added to the media as previously reported (Corwin et al., 1977). Cholesterol levels were analyzed using Ultraperformance Liquid Chromatography /Electrospray Ionization/Tandem Mass Spectrometry (UPLC/ESI/MS/MS) as previously reported (Ayciriex et al., 2012). PBS (110 μl) was added to sonicated samples. Extracts were spiked with an internal standard in methanol and saponified with 0.9 M NaOH. Saponified material was extracted with hexanes, derivatized with DMAPI in DMF, quenched and re-extracted with hexanes. Cholesterol levels were measured using a targeted UPLC-MS/MS method (Ayciriex et al., 2012). Semi-quantitative values are reported for cholesterol in μmol/g. Filipin staining detects intracellular cholesterol and lipids, and was undertaken as previously reported (Bornig and Geyer, 1974).

### Statistical analyses

For *in vitro* investigations, nonparametric comparisons were performed using the Mann-Whitney *U*-test. *P*<0.05 was considered significant.
